# Bone Marrow Aspiration and Biopsy in Critical Pediatric Patients: A Pathologist’s Perspective

**DOI:** 10.7759/cureus.17423

**Published:** 2021-08-24

**Authors:** Nilam Bhaskar

**Affiliations:** 1 Pathology, Employee State Insurance Corporation (ESIC) Hospital, Lucknow, IND

**Keywords:** bone marrow aspiration (bma), bone marrow biopsy (bmb), children, sternum aspiration, and posterior / anterior iliac crest aspiration

## Abstract

Bone marrow aspiration (BMA) and bone marrow biopsy (BMB), are medical modalities for the detection of non-malignant diseases as well as hematological malignancies in children. BMA attained momentum in the past few years owing to the possibility of achieving hematopoietic stem cells. Liquid bone marrow is aspirated through posterior/anterior iliac crest, tibia, and vertebral spinous process during BMA procedure in children for assessment of morphology at the microscopic level while BMB allows for cytological evaluation of marrow. It is also used for molecular genetics, immune-phenotypic, cytogenetics, and other specialized examinations. Additionally, BMA is also helpful in the reconstruction of tissue. These procedures should be performed by a specialist who has knowledge about the indication, contradictions, and hazards of these procedures due to their invasive nature. Still, there are no transparent guidelines available especially in the case of BMA for children. The purpose of this overview article is to focus on the specific guidelines to carry out the BMA and BMB in children and the techniques as well as complications associated with the BMA and BMB.

## Introduction and background

Bone marrow aspiration (BMA) is a technique to procure the marrow or core tissue of bone for diagnosis, examinations, or transplantation. In this procedure, a special needle is inserted into the bone, and marrow is collected through suction. BMA is required when different kinds of blood cells are affected, especially when all the three cell lines (white blood corpuscles, red blood corpuscles, and platelets) are affected which corresponds to the reduction in the production of bone marrow. Another familiar reason is to detect various lymph and myeloproliferative disorders or to know the staging of cancer [1-3], and additionally, it is required for transplantation. In spite of this, when infectious agents are present in the bone marrow, then a bone marrow culture is required for diagnosis. The most familiar location to obtain the bone marrow is the hip bone and the sternum is the second most familiar procuring site which remains hematopoietically active across the life. Due to this reason several hematologists use this site for obtaining marrow. The use of ileum is useful in the case of anxious patients of all ages particularly in adults and older children while the tibia is a satisfactory site in children including below the age of two years and neonates. A study led by Schaar et al., [4] showed that bone marrow aspiration from the iliac is difficult in small infants and some newborns. One of the common disorders that occur in children is acute ITP (acute idiopathic thrombocytopenic purpura). BMA is used with acute ITP in children to exclude parasites. Aspiration of cells from the bone marrow of children will occasionally produce critical knowledge which is not otherwise accessible [4-6]. More commonly, a Jamshidi type needle is utilized for bone marrow biopsy (BMB) in older children and adults [7]. However, such a needle available is a 14 gauge, 6 inch, and piercing instructions is about 2±2.5 cm into the bone and due to this reason, it is not suitable for neonates. The aim of this article is to provide information about clinical guidelines associated with BMA and BMB in children that may be helpful for pediatric hematologists & oncologists as well as for general pediatricians.

## Review

Clinical indications - bone marrow aspiration

Prenatal investigations of chromosomes are necessary to avoid the unnecessary investigation of bone marrow. It should be used for the investigation of particular conditions that are unsuited to life like Trisomy 18, 13, or Triploidy. BMA is not used for patients carrying Down syndrome [8]. The common clinical indications for BMA in children are as follows:

a) To evaluate the unusual peripheral blood finding, thrombocytopenia, unexplained anemia, and pancytopenia.

b) To investigate hypoplastic anemia, malignant hematological disorders, metastatic expansion of tumors, and congenital bone marrow deficiency syndrome [9-11].

c) To achieve the cultures of bone marrow especially in case of infection of unknown origin.

d) For evaluation of abdominal masses, lymphadenopathy, and hypersplenism.

e) To follow up the chemotherapy or transplantation of hematopoietic stem cells [11].

Clinical indication - bone marrow biopsy:

The common clinical indications for BMB in children are as follows:

a) Insufficient or failure of BMA.

b) Suspected BMF (bone marrow fibrosis).

c) Staging and evaluation of non-Hodgkin’s and Hodgkin’s lymphoma, and Ewing’s sarcoma as well as tiny blue oval cell tumors associated with childhood (rhabdo-myosarcoma, neuroblastoma).

d) Detection of myelodysplastic syndromes, aplastic anemia, and AML-M7 (acute megakaryoblastic leukemia) [12].

Contradictions

a) Hemorrhagic disorders (eg., hemophilia), distribution of intravascular coagulation, and concomitant employment of anticoagulants. If BMB or BMA is positively tested in the patients with the above-mentioned disease, then cessation of anticoagulation or for factor replacement must be considered prior to the procedure, and the patient must be carefully observed for 24 hours after the procedure. Serious thrombocytopenia is not an issue to BMA as sustained pressure is applied to the sampling site for the cessation of bleeding. For obese patients with severe thrombocytopenia in which a BMB is indicated then a platelet transfusion must be preferred to increase the platelet count by more than 15×109/L [11,12].

b) Infection of skin or earlier radiation therapy at the site of aspiration sampling.

c) Bone disorders like osteogenesis imperfecta or osteomyelitis.

Assessment of patients

a) Inspect the preliminary examination protocol to decide the requirement for the study and any other special needs (eg., cytogenetic or molecular studies and flow cytometry).

b) Consent must be obtained from the guardian of the child (13-18 years of age).

c) Evaluate the requirement for sedation (eg., general anesthesia or conscious sedation by use of inhaled nitric oxide (NO) or intravenous propofol). Children who weigh <10 kg and children with compromised respiration undergo this procedure in an operation room (children having higher mediastinal mass and those with serious respiratory discomfort must undergo an assessment of anesthesia prior to the procedure).

d) Inspect the platelet count in the children as well as the partial time of thromboplastin especially when the patient has a previous history of bleeding issues or is presently on anticoagulation therapy.

e) Investigate the skin at the aspiration site (bone marrow site) for signs of any infection.

f) Inspect the medical record for previous history of allergy especially to local anesthetics, anesthetic medications, or iodine solutions

g) Confirm that all necessary personnel are available (eg: laboratory technician for cytogenetic studies if needed).

h) Verify the identity of the patient.

i) Examine the anesthetic cream (topical one) to BMA sampling site up to 30-60 min prior to the procedure.

Local anesthesia

Pain is generated during the BMA or BMB procedure, hence it is not recommended to use these procedures without the employment of basal analgesia in children up to the age of 10 years. A local anesthetic solution is employed to numb the skin and periosteum at the procurement site. Lidocaine (or similar local anesthetic) is used as it is useful in the case of a patient who has no previous history of harmful reaction to the medication. During this procedure, 2 ml of 8.4% sodium bicarbonate solution and 8 ml of 1% lidocaine hydrochloride are taken out into a 10 mL syringe (1-1/2 inch needle with a 22 gauge). The sodium bicarbonate solution decreases the burning pain produced by the acidic lidocaine solution. Up to 2-5 ml of buffered lidocaine is employed to anesthetize a harshly annular area of the periosteum (approximately 1 cm in diameter). The adequacy of local anesthesia is checked by gently probing the periosteum with the help of a sharp point of the needle to recognize the remaining sensitive areas. If pain is experienced by the patient, then additional 1-2 ml of lidocaine is required to numb the remaining area of periosteum. Ketamine is also used to anesthetize the children. Rectal theopentone is another analgesia that is employed in the case of children based on the weight of the children (Table 1).

**Table 1:** Theopentone dose in children Dose of Theopentone in children according to their weight

Dose of Theopentone Drug (g)	Weight of the Children (Kg)
0.2	3.40- 5.67
0.3	5.71- 7.93
0.4	7.98-10.20
0.5	10.25-12.47
0.6	12.51-14.74
0.7	14.79-17.01
0.8	17.06-19.28
0.9	19.32-21.54
1	21.59-23.81

Techniques

The posterior superior iliac crest is a favorable site to procure the bone marrow from children as it possesses most of the cellular marrow [11] while the anterior iliac crest is a favorable site in the case of obese patients. If the children are < 18 months of age then anteromedial face of the tibial tuberosity is the favorable site for BMA [11]; moreover, this site does not yield sufficient specimens especially when this procedure is carried out by a doctor. In spite of this, another concern is the fracturing of the bone. Most of the hospitals utilized the posterior superior iliac crests for children as well as for small infants while for BMB in children; they preferred the posterior superior iliac crest. However, a technique associated with tibial tuberosity is used in the case of small neonates [13]. BMA and BMB should be carried out by trained doctors who have expertise in this technique [14]. The procurement of BMA or BMB samples is relatively simple to carry out if the periosteum of patients has been sufficiently anesthetized with procaine or lidocaine. BMA is carried out in children by using the following techniques:

a) Posterior Iliac Crest (PIC)/ Anterior Iliac Crest (AIC) Aspiration

An identical procedure is used for both PIC and AIC. A plastic syringe containing 1 ml anticoagulant (sodium heparin or any other anticoagulant) solution. Two tubes will be taken, one for the bone marrow study and another one for special studies like immune-phenotypic analysis, microbiologic culture, molecular analysis, and cytogenetic studies and this eliminates the requirement of marrow aspiration for special studies especially when unexpected results are achieved. After the preparation of marrow tubes, the sterile aspiration needle (Salah BMA needle) is received from a clinical scientist and carefully inspects the aspiration needle for manufacturing defects after removing the sternal guard and plastic guide. The aspiration needle is held in a horizontal position when the patient is present in the lateral decubitus position (for PIC) or supine position (for AIC) and keeps the index finger always at the tip of the aspiration needle to manage the depth of penetration. Several physicians create an incision of 0.2-0.3 cm in the skin by using a small scalpel blade for easy penetration of the aspiration needle. An incision is not required when the obturator is too sharp [15]. The aspiration needle is then injected with constant pressure. Prior to piercing the aspiration needle, the sufficiency of anesthesia at the site of injection is confirmed by interrogating the patient, then the needle is gently injected via the cortical bone by maintaining constant forward pressure and rotation. Decreased sensation of resistance basically indicates puncturing of the cortex and access of spongy trabecular bone by the aspiration needle which might cause an awful sensation in few patients. The aspiration needle is further pierced about 1 cm inside the cavity of bone marrow and after that stylet is unlatched and steadily removed followed by pulling the plunger associated with the syringe to aspirate bone marrow (approximately 0.3 mL). Aspirating bone marrow more than 0.3 ml leads to dilution of bone marrow with sinusoidal blood. Bone marrow is handed to the clinical laboratory scientist for smear formation. If spicules are observed in the smears then extra bone marrow is collected for special studies. If spicules are not detected in the smears then the procedure is repeated by piercing the bone marrow slightly deeper in contrast to the previous one. Another site should be pierced if the second attempt of aspiration is failed. If the third attempt is also failed then aspiration is done by using a 30 to 50 ml syringe. A sterile sponge is applied at the piercing site to stop the bleeding. A failure to receive a bone marrow fluid is called a "dry tap" which might be owing to the wrong positioning of the needle [16,17]. Repeated dry taps indicated the existence of Paget’s disease, aplastic anemia, hairy cell leukemia, myelofibrosis, marrow with a large number of leukemia cells, malignant lymphoma or metastatic tumor. Approximately 4-7% incidence of dry tap aspiration has been reported [11, 17-18]. BMB is necessary in case of a dry tap incident to know the nature of the pathological process [11,19]. Touch imprint is an admissible alternative of marrow smears to evaluate the morphological features especially when physicians are confronted with a dry tap.

b) Tibia

Tibia is another technique for bone marrow aspiration and biopsy in children [20,21] and comprises active marrow at its anterior end across the whole childhood life. Procurement of bone marrow can be successfully done from the tibia of children at 10 years of age. It is the usual practice to use the tibia as the primary site of puncture in all children under the age of two years. The insertion site is present on the superior flat non-muscular surface and the excellent point for this is up to 2 cm diagonally from the piercing site of the patellar tendon (the medial aspect of tibial tuberosity). If the aspiration needle is pierced at the lower end of the shaft, physicians confront difficulty due to progressive thickening of the cortex of the bone as well as thinning of the lumen.

c) Vertebral Spinous Process

The vertebral spinous process employed for BMA by laying down the children in the "lumbar puncture" posture. This approach utilized the tip of the spinous process in contrast to adults where the side of vertebrae is used. The spinal needles used for children ranging from one year to mid-childhood is 2.5 inches while 3.5 inches spinal needles are employed for older children. Local anesthesia (lidocaine or procaine) or topical anesthetic (EMLA cream) is used to numb the procuring site to circumvent the pain. In this procedure, children must be laid down in the lateral decubitus position by placing the pillow under the head to keep it in the same plane as the spine. The shoulder and hip must be positioned perpendicular with respect to the table and the lower back should be arched towards the doctors. Draw a line that connects the posterior superior iliac crest that will intersect the midline at around spinous process of L4 (4th lumbar vertebrae) while lumbar puncture in older children is carried out from L2-L3 interspace to L5-S1 interspace. Penetrate the needle through the skin and slowly progress the tip of the spinal needle at about 10 degrees cephalad (i. e. towards the umbilicus of patients). Remove the stylet and inspect for clear fluid that will flow into the needle after penetration of subarachnoid space. A “Pop” is felt during this procedure due to resistance produced by the ligament. Withdraw the spinal needle by leaving the tip, inspect for landmarks and gently progress the spinal needle again. Measure the opening pressure by using a manometer after attaching it to the spinal needle through a stopcock. Normal opening pressure ranges from 10-100 mmHg in young children while it ranges from 60-200 mmHg in older children (after eight years of age). Approximately 3-6 ml CSF (cerebrospinal fluid) fluid can be collected depending on the size of the child. Complications associated with this procedure include herniation, leakage of CSF fluid, pain, headache, bleeding, cardiorespiratory compromise, infection, and subarachnoid epidermal cyst. 

Bone marrow biopsy technique

BMB and BMA can be performed via the same site of the skin. Positioned the patients on the procedure table in the lateral decubitus position (for PIC) or supine position (for AIC). Hold the aspiration needle or biopsy needle (Jamshidi needle) with the anterior side and keep the index finger at the tip of the aspiration needle. After locking the stylet in place, an aspiration needle is injected through the skin of the patient pointing toward the anterior iliac spine. Using gentle pressure, advance the needle with a rotating motion until it is fixed with the bone. After removal of the stylet, the alternating motion of clockwise and counterclockwise are utilized then progress the aspiration needle gently for 2-10 mm inside the bone. The insertion depth is determined based on the size of the patient. Rotate the aspiration needle with 3 twists in the right direction and then into the left direction without progressing and this one more time. Withdraw the aspiration needle by applying a rotary motion. A spongy gauze pad is used to cease the bleeding from the aspiration site. A probe is employed from the caudal end to remove the specimen from the needle to avoid the crushing of samples. For sufficient biopsy in children, aspirate must contain approximately 0.5 cm of carefully preserved bone marrow [19].

Preparation

Bone Marrow Aspiration Smears

Preparation of smears from aspirate is a crucial part of the BMA and BMB procedure and it should be prepared immediately after the aspiration process to ensure a greater number of particles and to avoid impurity with peripheral blood. The traditional wedge technique is the common way to prepare peripheral blood smears. A drop of aspirate is put at one end of a sterile slide, and a second slide (spreader slide) is employed to spread the drop. The second slide is held at a 30-degree angle by keeping it in front of the aspirate drop and pulling this spreader slide until it touches the aspirate drop. Aspirate drop is permitted to spread up to the edge of the slide followed by pushing it forward at the 30-degree angle with a fast but steady motion. The particle crush method must be carried out by a skilled person in contrast to wedge preparation and this procedure is superior for morphologic investigations as it furnishes an ‘‘inside look’. The density of hematopoietic stem cells is too high and mast cells can be examined in the thick region [22]. In this procedure, a small aspirate drop is put at one end of a sterile microscopic slide and the second microscopic slide is held parallel with respect to the first just over the aspirate drop. Pressed the aspirate drop by the second slide by applying the gentle pressure and spread the aspirate drop by pulling the second slide across the whole length of the first microscopic slide. Coverslip smears are also done by the same method, but the handling of the coverslips is more tough and that is why it requires more experience but it provides superior morphology of aspirate drop of the bone marrow. In this procedure, 1-2 ml of the aspirate drop of bone marrow is kept on a Petri dish which is then tilted to exclude the blood and to increase the visibility of bone marrow particles [23]. One or two bone marrow particles are aspirated with the help of a Pasteur pipette and kept on cover glass (22 mm). Apply the gentle pressure on the first slide by using a second slide in the diagonal position to crush the particles, further pulling apart both coverslips to obtain smears [23] followed by placing the coverslips inside the labeled Petri dish until staining. Staining is performed manually by using forceps to grasp the coverslips during this process. Particle ‘‘clot’’ sections reflect the histologic sections of aspirate materials of bone marrow. The simplest clot section is prepared by drawing a little amount of aspirate material from bone marrow in a plain syringe and allowed to clot by itself. If clotting does not take place then add the little amount of thrombin solution drop by drop into the sample to induce the clotting process. The drawback of this procedure is that the aspirate particles present inside the clotted blood lead to difficult interpretation. Modification has been done to circumvent this problem which is described by Rywlin et al., [22,24]. In this modified method, a little amount of aspirate of bone marrow is kept into a tube containing ethylenediamine tetraacetic acid-an anticoagulant (EDTA). Further, this material is transferred into a tissue sampling bag. Particles are entangled into the bag while an excessive amount of blood filters through. The aspirate particles are fixed by using acetic acid-zinc-formalin (AZF) - a fixative agent followed by sectioning, and staining. One of the studies used in this procedure and showed that preparation of the histologic sections by this method is superior in contrast to the BMB for the investigation of lipid granuloma, nodular lymphoid hyperplasia, and lymphoid nodules [25]. The filtered blood is then centrifuged at a particular speed (rpm or g) remaining leads to the formation of four layers: fat layer and layer of peri-vascular cells, plasma layer, a layer of myeloid-erythroid cells (buffy coat), and red blood corpuscles (RBC) layer [26]. Measurement of the height of all layers produces information about the relative volume of every layer. Preparation of smears from the myeloid-erythroid and fat-peri-vascular layer is better for morphologic study because they are free from erythrocyte impurities. One of the studies also reported that the bone preparation of smears from buffy coats is superior to the aspirate of bone marrow for the examination of acid-fast bacilli [27]. Touch imprint preparations must be carried out from the BMB core when the aspirate sample is lacking marrow particles. In this procedure, the fresh core biopsy sample is kept on a sterile microscopic slide. Gentle pressure is applied on the biopsy core by using a second sterile microscopic slide and slightly rolled from side to side. At least two or three microscopic slides must be constructed with multiple imprints on every slide and cells present on the surface of the biopsy core attached to the slides. The imprint microscopic slides are stained with the Wright-Giemsa method for morphologic examination or employed for cytochemical investigation or immune-peroxidase staining. Several previous studies have shown the importance of biopsy imprints in the examination of malignancy [28-30]. Microscopic investigation of the smear associated with bone marrow is very helpful in the diagnosis of multi-plehaematologic disorders like iron (Fe) deficiency anemia, acute leukemia, and secondary deposits inside the bone marrow. Diseases of the reticuloendothelial system, thrombocytopenia, aplastic anemia, and many other disorders associated with blood. The ratio of myeloid-erythroid cells represents bone marrow activity. The ratio of myeloid: erythroid cell rises in case of leukemia and falls in case of leukopoiesis.

Complications

BMB and BMA are the salvage and safest procedures with a minimum risk of morbidity. Mortality is unfamiliar in the case of sternal aspiration while extremely uncommon in the case of BMA and BMB in association with the iliac crest. In the case of lethal sternal aspiration, several deaths have been reported due to pericardial tamponade from injury of the right ventricle or intra-pericardial aorta [22,31-33]. Pulmonary bone marrow emboli, growth of a sternal tumor mass, and sternal-manubrial separation are other unique complications of sternal marrow aspiration [34,35]. One of the studies reported the harmful events secondary to the investigation of bone marrow and the incidence of adverse events is 0.08% [36]. The most frequent harmful events were infection, hemorrhage, and consistent pain at the site of bone marrow. The bleeding episodes especially occurred at the retro-peritoneum, thighs, and buttocks and this condition is more common in the patients who underwent both BMA and BMB, or patients who were diagnosed with osteoporosis or myeloproliferative disorders [11,36]. In spite of this, patients with serious thrombocytopenia, coagulopathy, platelet dysfunction, renal impairment, von Willebrand’s disease, and obesity or patients who are receiving warfarin, heparin, or acetylsalicylic acid were also found at the high risk [37]. Breakage of the needle during the bone marrow aspiration is an extremely rare complication.

## Conclusions

Bone marrow aspiration and bone marrow biopsy are very useful procedures for the diagnosis and examination of hematological disorders. The hematopoietic activity of the sternum remains throughout life and is quickly available especially in a cooperative patient. The ileum aspiration has been extremely helpful in restless patients of any age particularly in adults and older children. The better puncture site for children less than two years of age is the tibia (upper end) and posterior iliac crest for older children. Standard operating clinical guidelines are mandatory for pediatric centers that are performing cone marrow aspiration and bone marrow biopsy. The recent development of advanced needles for bone marrow biopsy and these procedures are performed by skilled doctors may reduce the adverse events associated with bone marrow aspiration and bone marrow biopsy.
